# Klotho sensitive regulation of dendritic cell functions by vitamin E

**DOI:** 10.1186/s40659-016-0105-4

**Published:** 2016-11-24

**Authors:** Nguyen Thi Xuan, Phi Thi Thu Trang, Nguyen Van Phong, Nguyen Linh Toan, Do Minh Trung, Nguyen Duy Bac, Viet Linh Nguyen, Nguyen Huy Hoang, Nong Van Hai

**Affiliations:** 1 Institute of Genome Research, Vietnam Academy of Science and Technology, 18 Hoang Quoc Viet, Cau Giay, Hanoi, Vietnam; 2 Department of Pathophysiology, Vietnam Military Medical University, Ha Dong, Hanoi, Vietnam; 3 Department of Protein-Toxic-Cells, Biomedical & Pharmaceutical Applied Research Center, Vietnam Military Medical University, Ha Dong, Hanoi, Vietnam; 4 Vietnam Military Medical University, Ha Dong, Hanoi, Vietnam; 5 Institute of Biotechnology, Vietnam Academy of Science and Technology, 18 Hoang Quoc Viet, Cau Giay, Hanoi, Vietnam

**Keywords:** Dendritic cells, Klotho, LPS, Migration, ROS, Vitamin E

## Abstract

**Background:**

Dendritic cells (DCs) are the most potent professional antigen-presenting cells for naive T cells to link innate and acquired immunity. Klotho, an anti-aging protein, participates in the regulation of Ca^2+^ dependent migration in DCs. Vitamin E (VitE) is an essential antioxidant to protect cells from damage and elicits its inhibitory effects on NF-κB-mediated inflammatory response. However, the roles of VitE on mouse DC functions and the contribution of klotho to those effects both are unknown. The present study explored the effects of VitE on klotho expression, maturation, ROS production and migration in DCs.

**Methods:**

The mouse bone marrow cells were isolated and cultured with GM-CSF to attain bone marrow-derived DCs (BMDCs). Cells were stimulated with LPS (100 ng/ml) in the presence or absence of VitE (500 µM). RT-PCR and immunoprecipitation methods were employed to determine klotho expression, ELISA to determine cytokine release, flow cytometry to analyze number of CD86^+^CD11c^+^ cells, the intracellular expression of cytokines and reactive oxygen species (ROS) production and a transwell migration assay to trace migration.

**Results:**

Klotho transcript level and this hormone secretion in DC supernatant were enhanced by VitE treatment and further increased in the presence of NF-κB inhibitor Bay 11-7082 (10 µM). Moreover, VitE treatment inhibited IL-12p70 protein expression of, ROS accumulation in and CCL21-dependent migration of LPS-triggered mature DCs, these effects were reversed following *klotho* silencing.

**Conclusion:**

The up-regulation of klotho by VitE could contribute to the inhibitory effects of VitE on NF-κB-mediated DC functional maturation. The events might contribute to immunotherapeutic effect of VitE on the pathophysiology of klotho-related disease.

## Background

Klotho, a membrane protein, is predominantly expressed in the kidney, parathyroid glands and choroid plexus and to a lesser extent in the brain [1]. Klotho contributes to the suppression of aging phenotypes in humans and mice [2, 3]. The secreted form of KL has a putative sialidase activity that removes terminal sialic acids from N-linked glycans [4]. Thus, KL modulates the activities of multiple glycoproteins on the cell surface including calcium (Ca^2+^) channel [5]. Klotho elicits also inhibitory effects on nuclear factor-kappa B (NF-κB)-mediated inflammatory response [6, 7] and participates in the regulation of anti-oxidative defense [8]. The activation of NF-κB is triggered by ligation of a toll-like receptor (TLR)4 with its specific ligand, lipopolysaccharides (LPS) [9] and involves the phosphorylation of an inhibitory protein, IKB-α leading to transcription of multiple genes associated in the regulation of maturation/differentiation and cell survival [10]. Most recently, klotho expression has been shown in dendritic cells (DCs) [11], antigen presenting cells linking innate and acquired immunity [9].

The transformation from an immature to a mature DC is induced by LPS and characterized by upregulation of antigen-presenting and costimulatory molecules, release of inflammatory cytokines and accumulation of reactive oxygen species (ROS) [9, 12, 13]. The phenotypic and functional maturation of DCs are affected by Ca^2+^ influx, a crucial regulator of cell migration to draining lymph nodes (LNs) [9, 11, 14] and plays an important role in initiating the immune response by activating other immune cells. Following TLR4 ligation and subsequent Ca^2+^ entry, DCs migrate specifically into T cell areas of LNs, where they secrete chemokines that permit the attraction of naïve T cells and induce the proliferation and differentiation of antigen-specific T cells [9]. Both, Ca^2+^ entry into and migration of DCs are dependent on klotho expression and are virtually lacking in DCs isolated from *klotho*-deficient mice [11].

Vitamin E (VitE) is an essential fat-soluble antioxidant to protect cells from damage and elicits anti-inflammatory effect [15, 16] by preventing the pro-inflammatory cytokine synthesis such as tumor necrosis factor (TNF)-α and IL-12p70 [15, 17], which are important inducers of Th1 responses [15]. The immunosuppressive effects of VitE are mediated through NF-κB signaling pathway in human DCs [15]. Inhibition of this signaling results in suppression of DC-mediated immune response [18]. In addition, VitE has anti-carcinogenic effects, at least partially, by the stimulation of apoptosis of cancer cells [19, 20], although VitE has been shown to inhibit ROS production in these cells [21, 22]. Signaling mechanisms may contribute to the pro-apoptotic effects of VitE including phosphoinositide 3-kinase (PI3K)/Akt- [20], p53- and NF-κB [19] -dependent gene expression and caspase activation [23]. Thus, VitE deficiency is associated with increased risk of cancer and dysfunction of the immune system [24, 25]. Despite the numerous studies on anti-inflammatory and anti-carcinogenic activities of VitE, little is known about the roles of VitE on DC functions [15].

The present study has been performed to elucidate whether VitE influences the expression of klotho in DCs and whether klotho may contribute to the altered phenotypes of VitE-treated mature DCs. To this end, bone marrow derived mouse DCs (BMDCs) stimulated with LPS were treated with VitE in the presence or absence of *klotho* and the effects of VitE on the expression of co-stimulatory molecule CD86 in, the protein levels of pro-inflammatory mediators IL-12p70 and TNF-α of, ROS accumulation in and migration of DCs were determined.

## Results

### VitE regulated klotho expression through NF-κB signaling

**Fig. 1:**
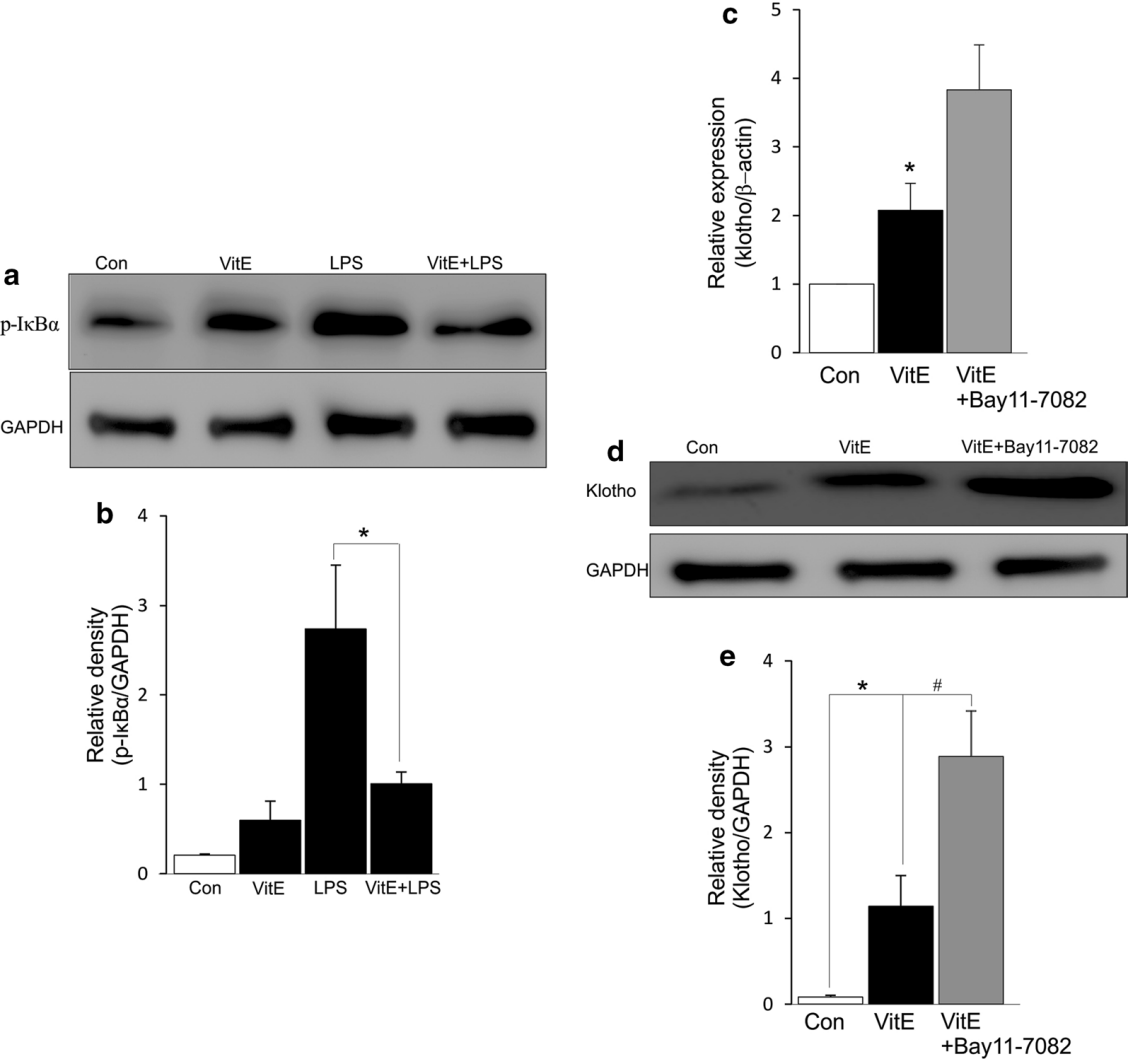
Effect of VitE on klotho expression. **a** Original Western blot of DCs were either treated with LPS (100 ng/ml) in the presence or absence of VitE (500 µM, 2 h) or left untreated (control). Protein extracts were analyzed by direct Western blotting using antibodies directed against p-IκBα and GAPDH. **b** Arithmetic mean ± SEM (n = 4) of the abundance of p-IκBα protein as the ratio of p-IκBα/GAPDH. **c** Arithmetic mean ± SEM (n = 5) of klotho transcript level is shown prior to control (*white bar*) and 5 h following incubation with VitE (500 µM) either in the absence (*black bar*) or presence of NF-κB inhibitor Bay 11-7082 (10 µM, *grey bar*). **d** Immunoprecipitation of klotho in DC supernatants is shown prior to control (*1st panel*) and 5 h following treatment with VitE (500 µM) either in the absence (2nd panel) or presence (*3rd panel*) of Bay 11-7082 (10 µM). Immunoprecipitates were stained for klotho and GAPDH antibodies. **e** Arithmetic mean ± SEM (n = 5) of the abundance of klotho protein as the ratio of klotho/GAPDH. *(p < 0.05) represent significant difference from control DCs, and ^#^(p < 0.05) indicates significant difference from VitE-treated DCs (ANOVA)

The activation of NF-κB signaling has been determined to be suppressed by treatment of cells with VitE [15]. To explore the modulation effects of VitE on NF-κB signaling in mouse DCs, bone marrow cells were cultured with GM-CSF for 8 days to attain BMDCs and subsequently treated with LPS (100 ng/ml) in the presence or absence of VitE (500 µM) for 2 h. In this study, LPS stimulation led to enhanced level of phosphorylated IκBα, the effect was significantly suppressed when VitE was present in the cell culture (Fig. 1a, b). Next, experiments were performed to examine the roles of VitE and NF-κB signaling on klotho expression. RT-PCR disclosed the upregulation of klotho mRNA expression following treatment of DCs with VitE for 5 h (Fig. 1c). Immunoprecipitation confirmed the expression of klotho protein in culture supernatant and revealed that the abundance of klotho protein was significantly enhanced by exposure of DCs to VitE (Fig. 1d, e). The further increase of klotho transcript and protein levels were observed by using pharmacological inhibition of NF-κB signaling pathway with Bay 11-7082 (10 μM, Fig. 1c–e). Thus, VitE participated in promoting klotho expression through suppressing activation of NF-κB signaling.

### Effect of VitE on klotho sensitive DC maturation

**Fig. 2:**
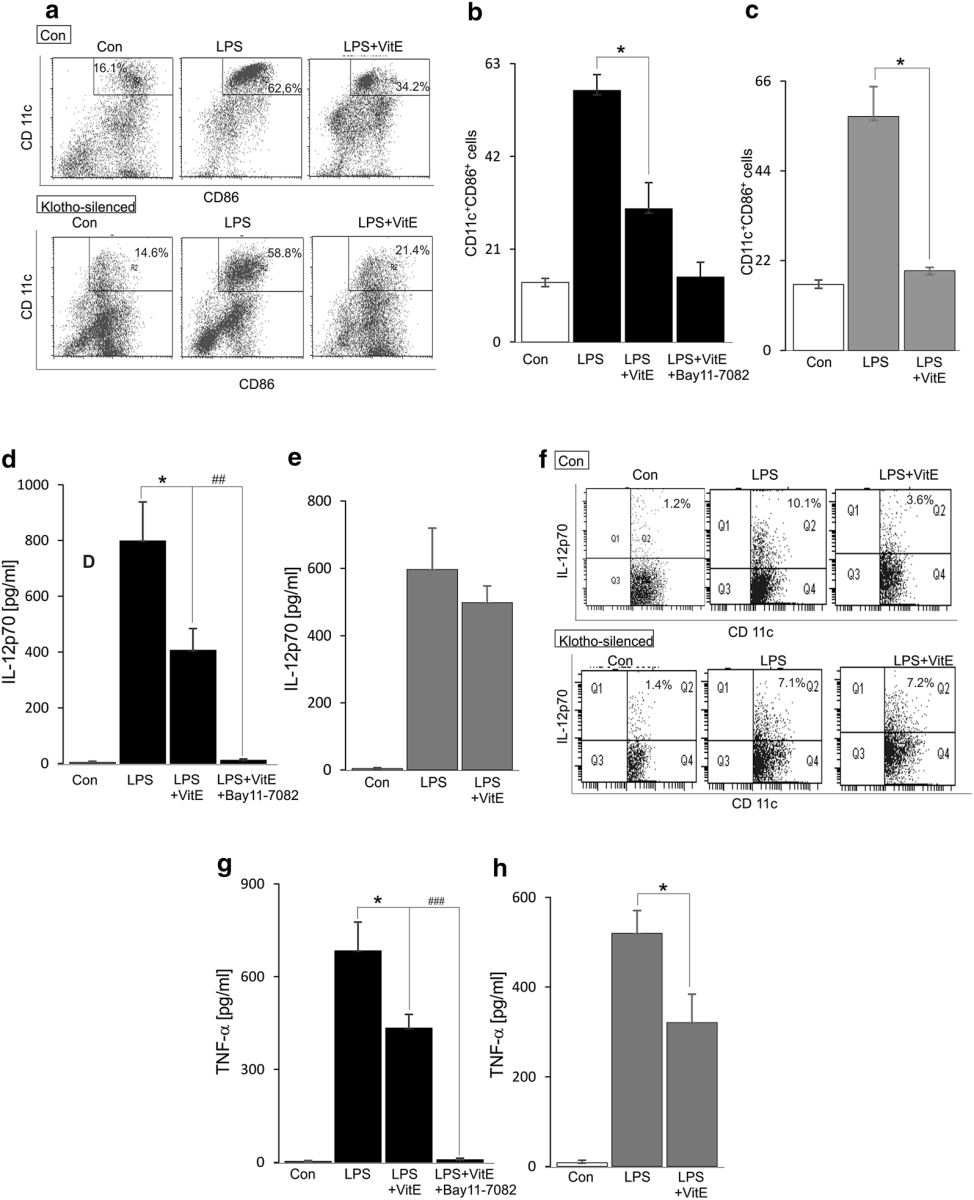
Effect of VitE on DC maturation. **a** Original dot plots representing the percentage of CD11c^+^CD86^+^ control-(*1st line*) and klotho-silenced (*2nd line*) DCs is shown prior to control (*1st panel*) and 24 h following treatment with LPS (100 ng/ml) either in the absence (2nd panel) or presence of VitE (*3rd panel*). **b** Arithmetic mean ± SEM (n = 5) of CD11c^+^CD86^+^ cells is shown prior to control (*1st panel*) and 24 h following treatment with LPS (100 ng/ml) either in the absence (*2nd bar*) or presence of VitE (*3rd bar*) or presence of VitE and Bay 11-7082 (10 µM) (*4th bar*). **c** Arithmetic mean ± SEM (n = 4) of CD11c^+^CD86^+^ cells transfected with *klotho* siRNA is shown prior to control (*1st panel*) and 24 h following treatment with LPS (100 ng/ml) either in the absence (*2nd bar*) or presence (*3rd bar*) of VitE. **d**, **g** Arithmetic mean ± SEM (n = 5–7) of IL12p70 and TNF-α production in DCs are shown prior to control (*white bar*) and 24 h following treatment with LPS (100 ng/ml) either in the absence (*2nd bar*) or presence of VitE (*3rd bar*) or presence of VitE and Bay 11-7082 (10 µM) (*4th bar*). **e**, **h** Arithmetic mean ± SEM (n = 5–7) of IL12p70 and TNF-α production in DCs transfected with *klotho* siRNA are shown prior to control (*1st bar*) and 24 h following treatment with LPS (100 ng/ml) either in the absence (*2nd bar*) or presence (*3rd bar*) of VitE. **f** Original dot plots representing the percentage of CD11c^+^IL-12p70^+^ control-(*1st line*) and klotho-silenced (*2nd line*) DCs is shown prior to control (*1st panel*) and 24 h following treatment with LPS (100 ng/ml) either in the absence (*2nd panel*) or presence of VitE (*3rd panel*). *(p < 0.05) represents significant difference from LPS-stimulated DCs; ^##^(p < 0.01) and ^###^(p < 0.001) indicate significant difference from LPS and VitE- treated DCs (ANOVA)

Since VitE contributes as an inhibitor of the inflammatory response via the NF-κB signaling pathway in human DCs [15], the roles of VitE on mouse DC maturation including the expression of co-stimulatory molecules and cytokine productions were examined. Treatment of the cells with VitE for 24 h significantly reduced number of CD11c^+^CD86^+^ cells (Fig. 2a, b) and the protein levels of IL-12p70 (Fig. 2d, f) and TNF-α (Fig. 2g) in LPS-stimulated DCs and the further decrease of those was seen by using pharmacological inhibition of NF-κB signaling pathway with Bay 11-7082 (Fig. 2b, d, g). Moreover, to determine whether the regulation of NF-κB-mediated DC functional maturation by VitE is dependent on klotho expression, DCs were transfected with *klotho* siRNA and followed by LPS treatment in the presence or absence of VitE for 24 h. Upon transfection with *klotho* siRNA, the inhibitory effects of VitE on number of CD11c^+^CD86^+^ cells and production of TNF-α in LPS-stimulated DCs were remained unaltered (Fig. 2a, c, h) whereas the protein level of LPS-induced IL-12p70 was unaffected in the presence of VitE (Fig. 2e, f). Interestingly, the inhibitory effect of VitE on the secreted and intracellular LPS-induced IL12p70 protein expression was indicated and these effects were abolished following klotho silencing (Fig. 2d–f). The evidence indicated that the upregulation of klotho contributed to the NF-κB-mediated inhibitory effect of VitE on the expression of IL-12p70 protein in DCs.

### Effect of VitE on klotho sensitive ROS formation

**Fig. 3:**
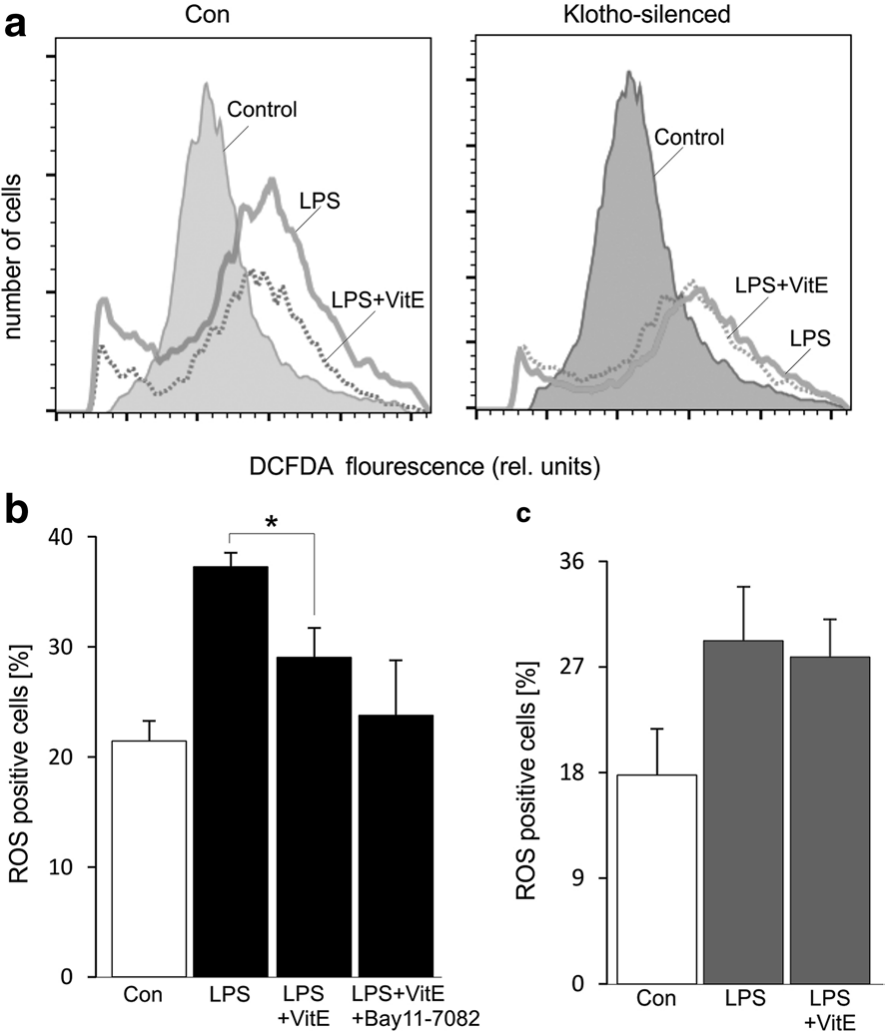
Effect of VitE on ROS formation. **a** Representative FACS histograms depicting ROS-dependent DCFDA fluorescence in control-(*1st panel*) and klotho-silenced (*2nd panel*) DCs are untreated (*gray filled*) or treated with LPS in the absence (*solid line*) or presence (*dotted line*) of VitE. **b** Arithmetic mean ± SEM (n = 5) of ROS production in DCs is shown prior to control (*white bar*) and 24 h following treatment with LPS (100 ng/ml) either in the absence (*2nd bar*) or presence of VitE (*3rd bar*) or presence of VitE and Bay 11-7082 (10 µM) (*4th bar*). **c** Arithmetic mean ± SEM (n = 5) of ROS production in DCs transfected with *klotho* siRNA is shown prior to control (*1st panel*) and 24 h following treatment with LPS (100 ng/ml) either in the absence (*2nd bar*) or presence (*3rd bar*) of VitE. *(p < 0.05) represents significant difference from LPS-stimulated DCs (ANOVA)

Treatment of DCs with LPS enhances ROS formation [13]. Several studies indicate the inhibition of ROS production by VitE [21, 26], it is consistent that the increase of LPS-triggered ROS production was significantly blunted by the presence of VitE (Fig. 3a, b). The effect tended to be further decreased by using NF-κB inhibitor Bay 11-7082. A recent study reveals that klotho plays a protective role of cells from the damage of oxidative stress induced by TNF-α [27]. Thus, additional experiments were performed to examine the effect of VitE on ROS accumulation in the absence of klotho. As noted in Fig. 3a and c, VitE treatment unaltered the increase of LPS-triggered ROS production when the cells were transfected with *klotho* siRNA, pointing out that the regulation of level of ROS by VitE was dependent on klotho expression in LPS-stimulated DCs.

### Effect of VitE on klotho sensitive DC migration

**Fig. 4:**
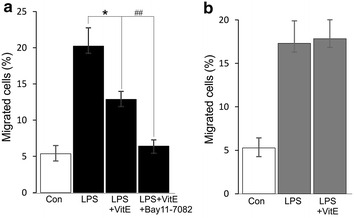
Effect of VitE on DC migration. **a** Arithmetic mean ± SEM (n = 5) of the normalized migration of DCs is shown prior to control (*white bar*) and 24 h following treatment with LPS (100 ng/ml) either in the absence (*2nd bar*) or presence of VitE (*3rd bar*) or presence of VitE and Bay 11-7082 (10 µM) (*4th bar*). **b** Arithmetic mean ± SEM (n = 5) of the normalized migration of DCs transfected with klotho siRNA is shown prior to control (*1st panel*) and 24 h following treatment with LPS (100 ng/ml) either in the absence (*2nd bar*) or presence (*3rd bar*) of VitE. *(p < 0.05) represents significant difference from LPS-stimulated DCs; ^##^(p < 0.01) indicates significant difference from LPS and VitE- treated DCs (ANOVA)

Recent study shows the role of VitE as a stimulator of Ca^2+^ influx [28] that participates in the regulation of DC functions including the migration [29]. Mature DCs migrate vigorously to CCR7 ligands, such as CCL21, which is expressed in lymph nodes [30]. Similar to our recent study, a transwell migration assay discloses a stimulatory effect of LPS on the migration of DCs [11], which was significantly blunted when the mature cells were treated with VitE (Fig. 4a). The effect was further decreased by using pharmacological inhibition of NF-κB signaling pathway with Bay 11-7082 (Fig. 4a). Furthermore, the LPS-induced migration of *klotho*-deficient DCs is inhibited [11]. In this investigation, we observed that the inhibitory effect of VitE on CCL21-dependent migration of LPS-matured DCs was blocked following klotho silencing (Fig. 4b), indicating that klotho was a mediator of NF-κB-dependent migration of VitE-treated mature DCs.

## Discussion

The present study demonstrated that VitE treatment resulted in suppression of klotho sensitive DC functional maturation. Accordingly, the LPS-induced increases in expression of IL-12p70 protein in, migration of and ROS production in VitE-treated DCs were significantly reduced, all effects were virtually abolished after suppressing klotho synthesis with *klotho* siRNA. Moreover, the effects are modulated through NF-κB activation [15, 31]. Therefore, this signaling could participate in regulation of klotho expression.

Klotho is a powerful regulator of Ca^2+^ entry into DCs [11] as illustrated that increased LPS-induced Ca^2+^ entry is blunted in DCs isolated from *klotho*-deficient mice [11] and subsequently modulates DC maturation and migration [9, 11, 14]. Importantly, klotho expression was upregulated in VitE-treated DCs and further increased by using pharmacological inhibition of NF-κB signaling pathway with Bay 11-7082 (Fig. 1c–e). Accordingly, VitE was expected to modify klotho sensitive NF-κB-mediated DC functions.

VitE seems to be an inhibitor of the maturation of DCs since all the upregulation of CD11c^+^CD86^+^ cells, the enhanced production of TNF-α and the increased levels of secreted and intracellular IL-12p70 proteins induced by LPS stimulation were inhibited in the presence of VitE and completely abolished by using pharmacological inhibition of NF-κB with Bay 11-7082 (Fig. 2). It is in agreement with a recent study that VitE plays an inhibitory role of NF-κB-triggered inflammatory response in human DCs [15]. In contrast, VitE induces NF-κB activation in cancer cells [19] and elicits anti-tumor effect by promoting the recruitment of tumor-specific effector T cells [32]. Interestingly, silencing of *klotho* by siRNA abolished the inhibitory effects of VitE on the levels of secreted and intracellular LPS-induced IL-12p70 proteins, but not TNF-α production and number of CD11c^+^CD86^+^ cells, suggesting that the downregulating effect of VitE on the level of IL-12p70 was dependent on klotho expression in DCs.

VitE has been shown to exert some antioxidant activity [19] and decrease oxidative stress-induced damage in endothelial and cancer cells [21, 26]. Both effects would be expected to inhibit ROS production in DCs. Several studies indicate the inhibitory role of VitE on ROS production leading to promoted cancer cell proliferation [21, 22], whereas others even consider VitE as an inducer of apoptosis of cancer cells via the regulation by intracellular signaling pathways [19, 20]. Therefore, the effect of VitE on the development of cancer is still in debate. The present observation, that VitE inhibited LPS-induced ROS accumulation in DCs, is similarly novel. The observations do not disclose the mechanisms underlying opposite effects of VitE on ROS production of cancer cells and DCs. Clearly, the signaling of suicidal death is different between DCs and cancer cells, as cancer cells grow and divide in an uncontrolled manner [33] whereas mature DCs are induced to undergo apoptosis [34]. In this study, the preventing effect of VitE on ROS production was again blunted in the presence of NF-κB inhibitor and completely abolished following *klotho* silencing (Fig. 3). Therefore, klotho expression could contribute to the downregulating effect of VitE on ROS accumulation upon LPS stimulation.

In addition, VitE has been also shown to suppress the migration in smooth muscle cells [35]. Similarly, we observed that the LPS-induced migration was downregulated when the cells were exposed to VitE (Fig. 4) and again reduced in the presence of NF-κB inhibitor Bay 11-7082. Our recent study revealed that DC migration is regulated by LPS-induced Ca^2+^ entry [11], which is abrogated in DCs isolated from *klotho*-deficient mice. Consistently, we indicated that the absence of klotho by treatment the cells with siRNA, the reduced migration in VitE-treated mature DCs was virtually reversed. The evidence pointed out the negative regulation of LPS-induced DC migration by VitE was sensitive to klotho expression.

## Conclusions

VitE enhanced the expression of klotho and subsequently impaired the protein expression of LPS-induced IL-12p70, ROS accumulation and migration, all effects were absent when klotho expression was downregulated in mouse DCs. The upregulation of klotho induced by VitE could contribute to the suppressing effects of VitE on NF-κB-mediated DC functional maturation. The effects of VitE are expected to affect the immune response.

## Methods

### Mice

Wild type pathogen-free BALB/c mice at the age of 6–8 weeks were purchased from Sigma-Aldrich (USA) and housed in a specific pathogen-free facility at Institute of Genome Research. The animals had free access to food and drinking water.

### Bone marrow-derived DCs

BALB/c mice were anesthetized with isoflurane gas and bone marrow cells were flushed out of the cavities from the femur and tibia with PBS. Cells were washed twice with RPMI-1640 and seeded out at a density of 4 × 10^6^ cells per 60-mm dish. Cells were cultured for 8 days in RPMI-1640 (GIBCO) containing: 10% fetal calf serum (FCS), 1% penicillin/streptomycin, 1% glutamine, 1% non-essential amino acids (NEAA) and 50 µm β-mercaptoethanol. Cultures were supplemented with GM-CSF (35 ng/mL, Sigma Aldrich, USA) and fed with fresh medium containing GM-CSF on days 3 and 6. Nonadherent and loosely adherent cells were harvested after 8 days of culture. Most (80% or more) of the cells expressed CD11c, which is a marker for mouse DCs. Experiments were performed on days 8–10. BMDCs were stimulated with LPS (100 ng/ml, Sigma-Aldrich) in the absence or presence of VitE (α-tocopherol, 500 µM, Sigma-Aldrich).

### Transfection of DCs with siRNA

*Klotho*-targeted siRNA (pre-designed siRNA, Applied Biosystems) was transfected into DCs (10^5^ cells/1 ml) with the help of Lipofectamine RNAiMAX Reagent (Invitrogen) according to the manufacturer’s recommendations. Cells were incubated for 48 h at 37 °C, 5% CO_2_. After washing three times with phosphate buffered saline (PBS), cells were used for experiments.

### Immunostaining and flow cytometry

Cells (4 × 10^5^) were incubated in 100 µl FACS buffer (PBS plus 0.1% FCS) containing fluorochrome-conjugated antibodies at a concentration of 10 µg/ml. The cells were stained with following antibodies (all from eBioscience): FITC-conjugated anti-mouse CD11c and APC-conjugated anti-mouse CD86. For intracellular cytokine staining of IL-12 p70, cells were stimulated with phorbol 12-myristate 13-acetate (50 ng/ml) and ionomycin (500 ng/ml, both from Sigma-Aldrich) for 3 h and followed by addition of brefeldin A (10 μg/ml, Sigma Aldrich) for another 4 h. The cells were then stained with APC-conjugated anti-mouse IL-12p70 (Thermo Fisher Scientific). After incubating with the Abs for 60 min at 4 °C, the cells were washed twice and resuspended in FACS buffer for flow cytometric analysis (FACSAria Fusion, BD Biosciences).

### RNA extraction and real-time RT-PCR

Total mRNA was isolated using the Qiashredder and RNeasy Mini Kit from Qiagen according to the manufacturer’s instructions. The SuperScript reverse transcriptase kit with oligo (dT) primers (Invitrogen) was used to generate cDNA. Quantitative RT-PCR for *klotho* and *β*-*actin* (Applied Biosystems) was performed on the Lightcycler 480 system (Roche). The ratio between the respective gene and corresponding β-actin was calculated per sample according to the ∆∆ cycle threshold method [36].

### Immunoprecipitation

DC supernatants were collected and stored at −80 °C until use for immunoprecipitation assay according to manufacturer’s instructions using a Protein G immunoprecipitation kit (Roche). Briefly, rat monoclonal klotho antibody (Ab) (Santa Cruz) was used at the concentration recommended by supplier for immunoprecipitation. DC supernatant was incubated with 50 μL of antibody-bound Protein G beads overnight at 4 °C in a thermomixer. Incubated beads were washed five times with a buffer containing 20 mM Tris–HCl, pH 7.5, 200 mM NaCl, 0.5% Triton X-100 and 2 mM EDTA. Immunoprecipitated proteins were then eluted by incubation at 95 °C for 5 min in 20 μl of 4× loading buffer (Roche) to attain the lysates.

### Immunoblotting

DCs (2 × 10^6^ cells) were washed twice in PBS, then solubilized in lysis buffer (Pierce) containing protease inhibitor cocktail (Sigma-Aldrich). Samples were stored at −80 °C until use for western blotting. The lysates were separated by 10% SDS-PAGE and blotted on nitrocellulose membranes. The blots were blocked with 5% nonfat-milk in triethanolamine-buffered saline (TBS) and 0.1% Tween-20. Then the blots were probed overnight with monoclonal antibodies directed against either p-IκBα or klotho or GAPDH (Cell signaling) diluted 1:1000 in blocking buffer, washed five times, probed with secondary antibodies (anti mouse or anti-rabbit, GE healthcare) diluted 1:5000 for 1 h at room temperature and washed final five times. Antibody binding was detected with the enhanced chemiluminescence (ECL) kit (Amersham). Densitometer scans of the blots were performed using Quantity One (BioRad).

### Cytokine measurement

TNF-α and IL-12p70 concentrations in DC culture supernatants were determined by using ELISA kits (eBioscience) according to the manufacturer’s protocol.

### Determination of ROS production

ROS production in DCs was determined utilizing 2′,7′-dichlorodihydrofluorescein diacetate (DCFDA). After the treatment, cells were collected and DCFDA (Sigma Aldrich) was added to the cell suspension at a final concentration of 10 µM. After 30 min of incubation in the dark at 37 °C, cells were centrifuged and the pellet was washed twice with ice-cold PBS. The pellet was then resuspended in FACS buffer and the fluorescence was analyzed with flow cytometry (FACSAria Fusion, BD Biosciences).

### DC migration assay

DCs were washed twice with PBS and suspended in RPMI 1640 medium. Migration was assessed in triplicate in a multiwell chamber with a pore diameter size of 8 µm (BD Falcon). The cell suspension (50,000 cells/ml) was placed in the upper chamber to migrate into the lower chamber in which either CCL21 (250 ng/ml, PeproTech) or medium alone as a control for spontaneous migration were included. The chamber was placed in a 5% CO_2_, 37 °C incubator for 4 h. The cells that migrated into the lower chamber were collected and counted under a light microscope.

The mean number of spontaneously migrated cells were subtracted from the total number of migrated cell and migration was considered by calculating the percentage of migrating cell related to input.

### Statistics

Data are provided as mean ± SEM, *n* represents the number of independent experiments. Differences were tested for significance using Student’s unpaired two-tailed *t* test or ANOVA. *P* < 0.05 was considered statistically significant.

## Abbreviations

BMDCs: bone marrow-derived dendritic cells
Ca^2+^: calcium
DCFDA: 2′,7′-dichlorodihydrofluorescein diacetate
ECL: chemiluminescence
GH: growth hormone
IL: interleukin
LPS: lipopolysaccharides
LNs: lymph nodes
NF-κB: nuclear factor-κB
p53: tumor protein p53`
PBS: phosphate buffered saline
PI3 K: phosphoinositide 3-kinase
ROS: reactive oxygen species`
TLR: toll-like receptor
TNF: tumor necrosis factor
TBS: triethanolamine-buffered saline
